# Two-dimensional titanium carbide MXene produced by ternary cations intercalation via structural control with angstrom-level precision

**DOI:** 10.1016/j.isci.2022.105562

**Published:** 2022-11-15

**Authors:** Zehai Xu, Yufan Zhang, Minmin Liu, Qin Meng, Chong Shen, Lushen Xu, Guoliang Zhang, Congjie Gao

**Affiliations:** 1 Center for Membrane and Water Science & Technology, Institute of Oceanic and Environmental Chemical Engineering, State Key Lab Breeding Base of Green Chemical Synthesis Technology, Zhejiang University of Technology, Hangzhou 310014, P. R. China; 2 College of Engineering, Carnegie Mellon University, Pittsburgh, PA 15213, USA; 3 College of Chemical and Biological Engineering, State Key Laboratory of Chemical Engineering, Zhejiang University, Hangzhou 310027, P. R. China

**Keywords:** Nanomaterials, Materials structure

## Abstract

Highly effective decontamination of lead is a primary challenge for ecosystem protection and public health. Herein, we report a methodology of ternary cations intercalation to synthesize Ti_3_C_2_T_x_ MXene by structural control with angstrom-level precision through mixed fluorinated salts wet etching-alkalization approach for high-efficient lead adsorption. The successive introduction of lithium, potassium, and sodium ions continuously weakens interaction forces between Ti_3_C_2_T_x_ layers, resulting in achieving fine tailored interlayer distance from 9.8 to 15.9 Å. A high density of complexing groups are formed after ternary cations intercalation, which greatly improve the hydrophilicity of Ti_3_C_2_T_x_ to enhance the accessibility and shorten the mass transfer and provide abundant adsorption sites to exhibit strong complexing effects with lead ions. The prepared ternary cations-intercalated Ti_3_C_2_T_x_ nanosheets exhibited a high adsorption capacity (267.2 mg/g) toward lead ions and sharply cut down lead concentration from 10 to 0.009 mg/L, far below the drinking water standards (0.015 mg/L).

## Highlights

• Ti_3_C_2_T_x_ MXene was achieved by mixed fluorinated salts wet etching-alkalization
• Structural control with angstrom level was realized by ternary cations intercalation
• High density of complexing groups exhibited strong complexing effects with lead ions
• Yield of generated Ti_3_C_2_T_x_ was greatly enhanced compared with single Li^+^ intercalation

## Graphical abstract

**Figure:**
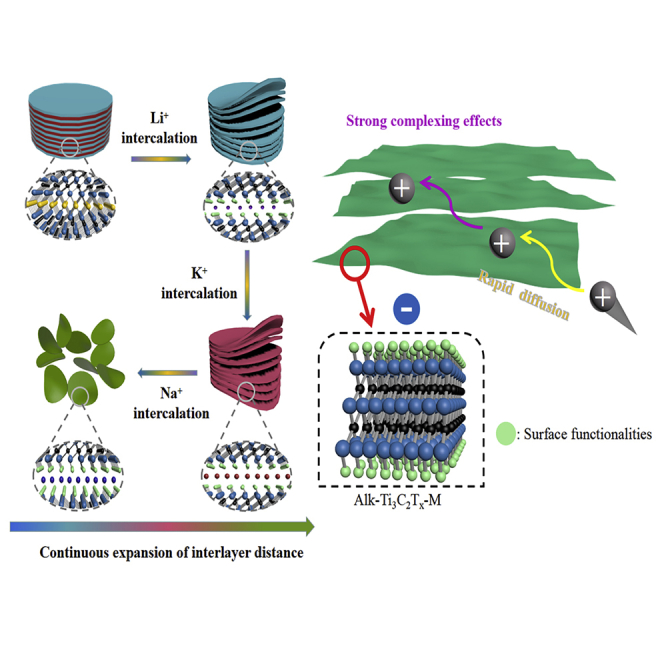

## Introduction

Heavy metal pollutions have long been serious public threats to environment and health, which cause dreaded diseases in human beings and other biomes, nerve damage, and birth defects.^1^^,^^2^^,^^3^^,^^4^ In particular, lead is a priority target for the United States Environmental Protection Agency due to its high degree of toxicity and prevalence.^5^ Major sources of lead pollution contain building materials, oil refining, lead-acid batteries, and paints, which are primary issues of concern to developed and developing countries. Among numerous technologies developed over decades for heavy metal removal, the adsorption takes the advantages of low cost, simple operation, high efficiency, reuse, possible regeneration, and easy industrialization.^6^^,^^7^^,^^8^^,^^9^^,^^10^ So far, many materials have been investigated for Pb(II) adsorption including activated carbon, alumina microsphere, hydrotalcite, silicon sphere, molecular sieve, and zeolite. However, conventional adsorbents are usually much limited because of poor competitive adsorption performance, low adsorption capacity, and unstable regeneration ability. Therefore, design and preparation of new nanostructured adsorbents with high adsorption capacity for toxic lead remain a great challenge.

Being a new type of thriving two-dimensional materials, MXenes are etched from a family of ternary-layered metal carbides, nitrides, and carbonitrides MAX phase since 2011,^11^ which have high specific surface area, abundant functional groups onto the surface including fluorine (-F), hydroxyl groups (-OH), and other oxygen-containing groups and developed pore structure. Theoretical calculations and experiments demonstrated that MXenes had excellent adsorption properties toward heavy metal ions.^12^^,^^13^^,^^14^^,^^15^ For example, the theoretical adsorption capacity of Ti_3_C_2_ MXene for Pb may be calculated as high as 1920 mg/g by density functional theory and molecular dynamics simulation.^16^ Besides theoretical calculations, 2D alk-MXene synthesized by alkalization intercalation displayed preferential lead ions sorption behavior with an excellent lead ion adsorption capacity around 140 mg/g.^12^ Despite the progress achieved, the narrow spacing between layers in lamellar-stacked structure hinders the generation of active adsorption sites on the surface and reduces ions transportation and diffusion, thus seriously preventing the contact between adsorbents and adsorbates.^17^ To solve this problem, intercalating alkali metal ions such as Li+, K^+^, Na^+^, and Mg^2+^ was attempted to enlarge the interlayer spacing.^18^^,^^19^ Due to the intercalation of cations between the Ti_3_C_2_T_x_ layers, the interlayer adhesion was weakened, multilayered Ti_3_C_2_T_x_ could be delaminated in subsequent sonication or oscillation.^20^^,^^21^ Nevertheless, only by single cation intercalation, the goal of high-efficient stratification and tunable interlayer distance cannot be reached. In particular, the increments of interlayer distance are very limited due to small size of single metal ions, resulting in unsatisfying adsorption performance. These facts suggest that the key to the bottleneck lies in the rational design of MXene nanosheets with wide and controlled interlayer spacing.

As noticed, Ti_3_C_2_T_x_ MXene nanosheets may show specific response to different cations in increasing the interlayer distance.^22^ In general, metal ions with small diameter are easier to insert into the Ti_3_C_2_T_x_ layers than ions with large diameter while ions with large size have a pronounced effect on enhancing interlayer spacing. The interfacial properties and terminal groups on the MXene interface can be obviously changed by forming new chemical bonding between MXene nanosheets and intercalated cations.^23^^,^^24^^,^^25^ In this case, if multiple kinds of metal ions with different diameter are successively introduced during the fabrication process, the interfacial properties and spacing of MXenes nanosheets can be rational designed due to the ion intercalation synergistic effect. According to the above observations, we envisage that Ti_3_C_2_T_x_ MXenes nanosheets with precisely controlled interlayer spacing at Å-size should be of great interests in wide fields; however, a clear and practical demonstration of such is still lacking.

**Figure 1:**
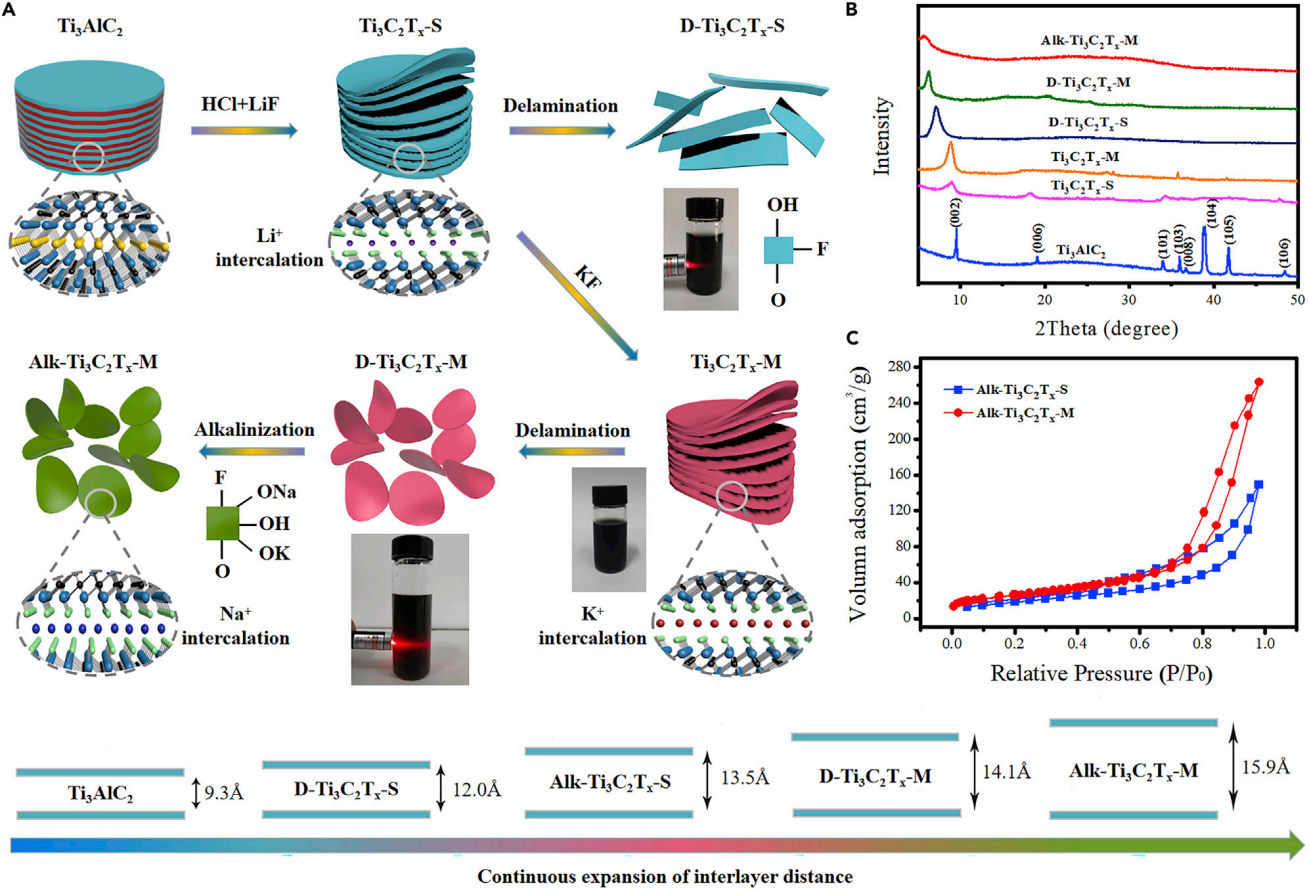
Scheme of fabrication of ternary cations-intercalated Ti_3_C_2_T_x_ MXene nanosheets with angstrom-level precise control of interlayer distance (A) Two-dimensional Ti_3_C_2_T_x_ MXene nanosheets with controlled interlayer spacing at angstrom-level precision were realized by ternary cations intercalation method. The photographs of delaminated Ti_3_C_2_T_x_ solutions displayed obviously Tyndall scattering effect. (B) XRD patterns of Ti_3_AlC_2_, Ti_3_C_2_T_x_-S, Ti_3_C_2_T_x_-M, D-Ti_3_C_2_T_x_-S, D-Ti_3_C_2_T_x_-M, and Alk-Ti_3_C_2_T_x_-M. (C) Adsorption/desorption isotherms of Alk-Ti_3_C_2_T_x_-S and Alk-Ti_3_C_2_T_x_-M.

Here, we report a methodology for realizing the structural control with angstrom-level precision in facile synthesis of active two-dimensional titanium carbide (Ti_3_C_2_T_x_) MXene based on ternary cations intercalation (Figure 1). Our strategy contains three main concepts: (i) Successive and complete incorporation of cations with various ratios into two-dimensional Ti_3_C_2_T_x_ MXene endowed MXene nanosheets with much -OH, -ONa, and -OK groups, generating tunable electrostatic interactions between layers to exercise full and precise control of interlayer distance at angstrom level and high surface area. (ii) Formation of a high density of strong lead complexing groups including -ONa and -OK during ternary cations intercalation to render high affinity for lead ions and efficient utilization of lead binding sites; (iii) Strong metal-ligand interaction with lead ions was realized by efficiently utilizing lead binding sites to achieve high sorption selectivity. The highly controlled interlayer spacing was obtained through two-step mixed fluorinated salts wet etching-alkalization strategy. In comparison to those Ti_3_C_2_T_x_ nanosheets etched by single fluorinated salt, abundant adsorption sites were generated due to the high surface area of Ti_3_C_2_T_x_ nanosheets produced through mixed fluorinated salts wet etching, which made surfaces of Ti_3_C_2_T_x_ nanosheets are highly accessible to heavy metal ions. In addition, large numbers of oxygen functional groups were produced to enhance the hydrophilcity and facilitate water flow. The synthesized ternary cations-intercalated Ti_3_C_2_T_x_ MXene nanosheets achieved high adsorption capacity toward lead ions for water purification and sharply cut down lead concentration from 10 mg/L to extremely low 0.009 mg/L, which was far below the drinking water standards (0.015 mg/L).

## Results and discussion

The fabrication of ternary cations-intercalated Ti_3_C_2_T_x_ MXene nanosheets is briefly summarized in Figure 1A; the bulk Ti_3_AlC_2_ was firstly etched by LiF and HCl to eliminate atomic Al layer in Ti_3_AlC_2_ phase and obtain multilayer Ti_3_C_2_T_x_ nanosheets. Subsequently, the sample was deeply etched after the introduction of KF; the extra fluoride ions were more easily to attack the inside of MAX phase and weaken the van der Waals force between layers, thus obtaining more single or double-layer Ti_3_C_2_T_x_ MXene nanosheets (Ti_3_C_2_T_x_-M) with the assistance of ultrasonic treatment compared with single fluorinated salt-based etching method. The Tyndall scattering effect can be clearly observed in the delaminated Ti_3_C_2_T_x_-M (D-Ti_3_C_2_T_x_-M) colloidal suspension. After the delamination, the prepared Ti_3_C_2_T_x_ nanosheets etched by mixed fluorinated salts were alkalized to achieve abundant -OH/-ONa/-OK complexing groups and introduced sodium ions to further broaden interlayer spacing. The interaction forces between layers can be gradually weakened by successively introducing ternary cations to realize the precise control of interlayer structure.

In order to confirm the precise control of interlayer spacing of Ti_3_C_2_T_x_ nanosheets by ternary cations intercalation method, we performed X-ray diffraction (XRD) for Ti_3_C_2_T_x_ nanosheets intercalated with different cations. From the XRD patterns (Figure 1B), the diffraction peak around 39° which corresponded the (104) crystal facet disappeared after etching process, which further confirmed that the atomic layer of Al was selective removed. The diffraction peak of (002) facet shifts to a lower angle, indicating the increment of interlayer spacing of Ti_3_C_2_T_x_ after etching.^26^^,^^27^ The d_c/2_ (half of the c-lattice parameter) of D-Ti_3_C_2_T_x_-S intercalated with single cation was enhanced from 9.8 Å to 12.0 Å after delamination while D-Ti_3_C_2_T_x_-M intercalated by dual cations showed a d_c/2_ of 14.1 Å, the interlayer spacing increased 2.1 Å after introducing KF. The results demonstrated that the increase of interlayer distance was attributed to that the introduction of potassium ions which inserted the interlayers of Ti_3_C_2_T_x_ nanosheets endow MXene nanosheets with -OK groups and obviously enhance the zeta potential value of D-Ti_3_C_2_T_x_-M (−32.8 mV) compared to D-Ti_3_C_2_T_x_-S (−25.6 mV) (Figure S1), thus generating strong electrostatic interactions between layers. After the introduction of sodium ions, the diffraction peak of (002) facet of Ti_3_C_2_T_x_ nanosheets intercalated ternary cations shifted to a lower angle and the d_c/2_ increased to 15.9 Å; this was due to that stronger electrostatic interactions were formed after alkalization approach, consequently further broadening the spacing. The size control of samples can provide a good opportunity to acquire satisfactory physical and chemical properties.^28^^,^^29^ Owing to the increase of interlayer spacing, the ternary cations-intercalated D-Ti_3_C_2_T_x_-M nanosheets (Alk-Ti_3_C_2_T_x_-M) achieved a high surface area of 92.6 m^2^ g^−1^ (Figure 1C), which was much higher than that of Alk-Ti_3_C_2_T_x_-S (72.1 m^2^ g^−1^) and most reported existing Ti_3_C_2_T_x_ MXene-based adsorbents. The pore size distributions of Alk-Ti_3_C_2_T_x_-S and Alk-Ti_3_C_2_T_x_-M samples demonstrated that the prepared MXene remained uniform pore structure after the ion intercalation (Figure S2). The enlargement of surface area and interlayer spacing caused is beneficial to acquire abundant active sites for adsorption.

**Figure 2:**
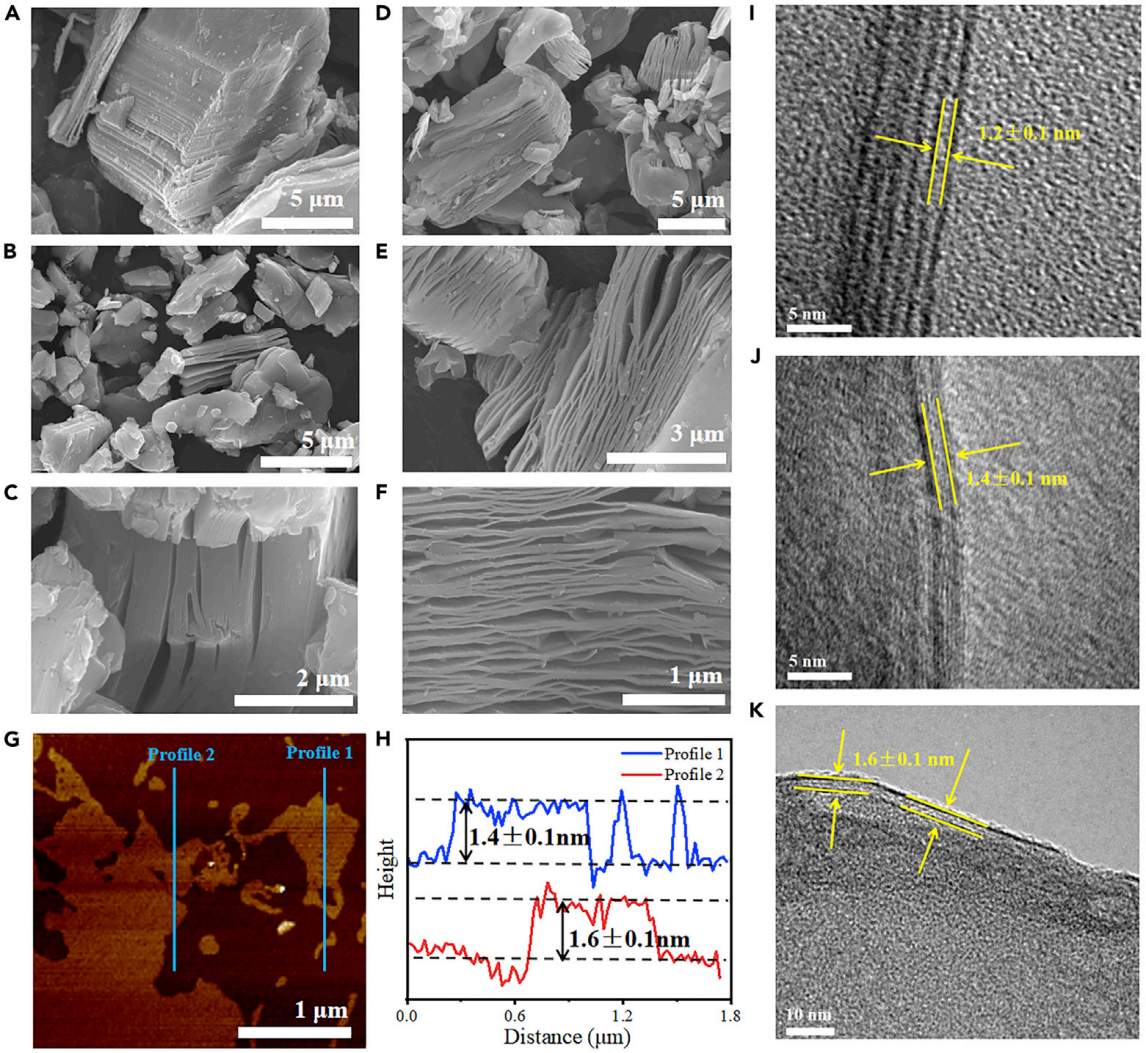
Morphology and microstructure of cation-intercalated Ti_3_C_2_T_x_ nanosheets (A−F) SEM images of bulk Ti_3_AlC_2_ (A), Ti_3_C_2_T_x_-S (B,C), and Ti_3_C_2_T_x_-M (D, E, F) at different magnifications. (G and H) AFM image and corresponding height profiles of D-Ti_3_C_2_T_x_-M. Data are represented as mean ±. HRTEM images of D-Ti_3_C_2_T_x_-S (I), D-Ti_3_C_2_T_x_-M (J), and Alk-Ti_3_C_2_T_x_-M (K) samples. Data are represented as mean ± HRTEM.

To evaluate the difference in morphology of prepared Ti_3_C_2_T_x_ nanosheets intercalated by single, dual, and ternary cations, we first took single fluorinated salt (LiF) to selectively etch Al atom layer in bulk Ti_3_AlC_2_ to obtain multilayered Ti_3_C_2_T_x_ (labeled Ti_3_C_2_T_x_-S) (Figure 2A); however, the accordion-like structure was not obvious (Figures 2B and 2C). When the KF was introduced to the etching reaction (LiF:KF = 5:1), it was found that the Ti_3_C_2_T_x_ nanosheets intercalated by dual cations (labeled Ti_3_C_2_T_x_-M) exhibited obvious accordion-like structure (Figures 2D–2F). The elimination of the Al atom layer was evidenced by EDX (Table S1), almost no Al was discovered Ti_3_C_2_T_x_-M in sample, which indicated that the Al atom layer in bulk Ti_3_AlC_2_ phase was destroyed by regular etching. Atomic force microscope (AFM) was used to measure the thickness of MXenes, and the synthesized delaminated Ti_3_C_2_T_x_-M (D-Ti_3_C_2_T_x_-M) possessed a thickness of ∼1.5 nm (Figures 2G and 2H). Since the lithium ions and potassium ions sufficiently inserted into the interlayer between of the multilayered Ti_3_C_2_T_x_-M, interlayer forces between layers were weakened, resulting in numerous single or double layered nanosheets were peeled off under strong shear force. Moreover, the effects of molar ratio and etching temperature on morphology were investigated. The unstratified structures which were like the original Ti_3_AlC_2_ indicated the poor etching effect when the molar ratio of LiF:KF was 3:1. After adjusting the molar ratio of LiF:KF to 7:1, an obvious accordion structure appeared again (Figure S3). From the SEM images of multilayered Ti_3_C_2_T_x_-M synthesized at different temperatures, it can be found that the etching effect is not obvious at 30°C; there was only a small gap between layers. When the etching temperature increased to 35°C and 40°C, the layered structure separated very well from each other, demonstrating a better etching effect operated at 35°C and 40°C.

Microstructure of prepared Ti_3_C_2_T_x_ nanosheets was analyzed by TEM and high-resolution TEM (HRTEM) analysis. After the delamination, many few-layered delaminated Ti_3_C_2_T_x_-S (D-Ti_3_C_2_T_x_-S) nanosheets still remained in the suspension (Figure S4) while ultrathin structure with single- or double-layered nanosheets was observed in D-Ti_3_C_2_T_x_-M sample (Figure S5). The HRTEM image displayed the lattice spacing of 0.105 nm, which attributed to (002) crystal facet of Ti_3_C_2_T_x_ (Figure S5).^11^^,^^21^^,^^30^ Moreover, Figure S5 exhibited the cross-section image of double-layered D-Ti_3_C_2_T_x_-M nanosheets; three Ti monolayers were obviously detected with distance of 0.72 nm, which was similar to the reported literature.^31^ Subsequently, the D-Ti_3_C_2_T_x_-M was intercalated with sodium ions by alkalinization; ternary cations-intercalated functionalized nanosheets were generated. As depicted in HRTEM images (Figures 2I–2K), the interlayer spacing was gradually increased from 1.2 to 1.6 nm after cations intercalation, which is in agreement with XRD analysis. The alkalized D-Ti_3_C_2_T_x_-M (Alk-Ti_3_C_2_T_x_-M) sample remained the similar structure with a thickness of ∼1.40 nm; the ultrathin ternary cations-intercalated Ti_3_C_2_T_x_ MXene nanosheets can provide enough contact with adsorbates, consequently improving the adsorption performance. The change in composition was also evaluated by EDX analysis (Figure S6 and Table S1); the atomic ratio of O/F increased after alkalinization, revealing the oxygen functional groups (-OH/-ONa/-OK) replaced F site.

In general, the etching degree of MAX under various experimental conditions was objectively represented by the exfoliation efficiency of MXenes. The concentration of D-Ti_3_C_2_T_x_-M suspension and the exfoliation efficiency was calculated by vacuum filtration method. The calculated yield was based on exfoliation efficiency and the formula is listed as follows:

$$Yield\;\left(\%\right)=\frac{M_{dispersed\;MXene}}{{M\;}_{multilayer\;MXene}}$$

As illustrated in Table S2, when the molar ratio of LiF:KF was 3:1, the yield (38.9%) was slightly lower than that of sample prepared by using LiF as etching agent (43.7%). It was due to the extra K^+^ dominated the etching process and generated large steric hindrance, thus hindering the reaction in Ti sites inside and causing poor etching performance. Nevertheless, when the content of KF reduced, the high yield can be achieved. In particular, the yield can reach 62.9% with a LiF:KF molar ratio of 5:1 at 35°C, which was much higher than the value in reported literature (45%).^21^ In particular, the percentage of single- and double-layered Ti_3_C_2_T_x_ nanosheets can reach 90% in the whole dispersed Ti_3_C_2_T_x_, which can be observed in AFM image at low magnification (Figure S7). It can be attributed to the generation of wider fluoride ion channels created by the addition of KF. Therefore, the added fluoride ions were more easily to attack the inside of MAX phase and etched deeply. Moreover, the interlayer van der Waals force was weakened, thus easily obtaining single- or double-layered Ti_3_C_2_T_x_ nanosheets. In addition, the influence of temperature on the etching effect was analyzed and fully verified in Table S2. On account of the results above, the etching effect was not satisfactory at 30°C; the yield was only 18.2%. When the temperature enhanced to 40°C, the yield was slightly reduced (60.2%). The results reflected that the mixed fluorinated salts-based wet etching strategy had good potential for the synthesis of delaminated MXene.

The surface chemical state of cation intercalated Ti_3_C_2_T_x_ nanosheets is of importance for adsorption which follows to the principles of coordination chemistry and electrostatic attraction. The absorption peak in Fourier transform infrared (FTIR) spectra at 1796 cm^−1^ can be assigned to a stretching vibration from a carbonyl group or a conjugated carbonyl group, which derived from a terminal group at the edge of the Ti_3_AlC_2_ phase.^32^^,^^33^ After etching process, the absorption peaks at 1616 cm^−1^ and 3420 cm^−1^ were attributed to the stretching vibration of -OH, which was derived from the water molecules bound between the Ti_3_C_2_T_x_ sheets and the surface hydroxyl functional groups of Ti_3_C_2_T_x_ nanosheets (Figure S8).^34^ No new absorption peaks appeared after stripping alkalinization, indicating that the two-step operation did not destroy the surface functional groups of original Ti_3_C_2_T_x_. The appearance of the characteristic peaks of K 2p and Na 1s in full X-ray photoelectron spectroscopy (XPS) spectra indicated that potassium and sodium ions were bound to insert into prepared MXenes (Figure S9). More importantly, the relative changes in the peak proportion of O 1s and F 1s were consistent with the EDX and FTIR results, indicating that the oxygen-containing functional group replaced -F after alkalization. The intensity of O 1s peak in Alk-Ti_3_C_2_T_x_-M was higher than that of Alk-Ti_3_C_2_T_x_-S sample, revealing more oxygen functional groups were generated by using mixed fluorinated salts-based wet etching strategy. After the alkalization, some new peaks of Ti 2p appeared in the spectra. The C 1s spectra of Alk-Ti_3_C_2_T_x_-M displayed 4 kinds of chemical states of carbons, including C-Ti (281.8 eV), C-C (284.9 eV), C-O (286.4 eV), and O=C-O (288.8 eV).^12^ The curves of O 1s spectra can be divided into five peaks. The binding energy at 530.1 eV is the peak of the Ti-O bond, which is the main form of the bond of O, and the binding energy at 530.8, 532.0, 533.3, and 535.3 eV can be assigned to the C-Ti-O_x_, C-Ti-(OH)_x_, O-C, and H_2_O_ads_, respectively.^6^ It is worth noting that the content of C-Ti-(OH)_x_ (23.3%) in Alk-Ti_3_C_2_T_x_-M is higher than that of C-Ti-(OH)_x_ (19.2%) in Alk-Ti_3_C_2_T_x_-S sample, indicating more fluorine groups are superseded by -OH, -ONa, and -OK groups which was evidenced by EDX analysis (Figure S10). The presence of Ti-OH, Ti-ONa, and Ti-OK groups can improve ion exchange behaviors in nature and display strong interactions with heavy metal ions.^12^

**Figure 3:**
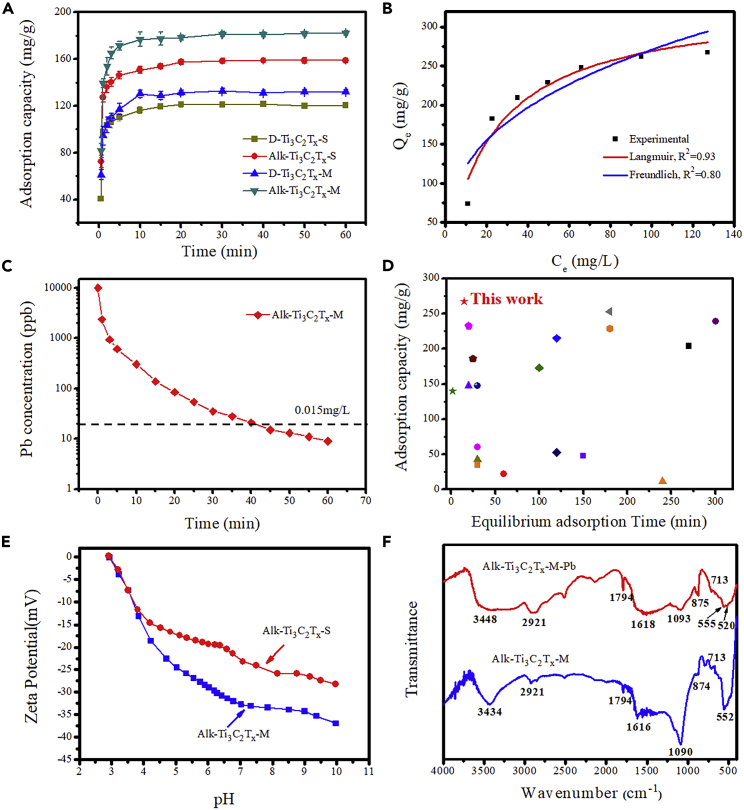
Performance of ternary cations-intercalated Ti_3_C_2_T_x_ nanosheets in removal of lead ions (A) Comparison of adsorption performance of D-Ti_3_C_2_T_x_-S, D-Ti_3_C_2_T_x_-M, Alk-Ti_3_C_2_T_x_-S, and Alk-Ti_3_C_2_T_x_-M. (B) Adsorption isotherm of lead ions. (C) Lead adsorption kinetics of Alk-Ti_3_C_2_T_x_-M under the lead initial concentration of 10 mg/L. (D) Adsorption performance of state-of-the-art for lead adsorption in literature. (E) Zeta potential of prepared samples. (F) FTIR spectra of Alk-Ti_3_C_2_T_x_-M sample before and after adsorption of lead ions.

To explore the relationship between adsorption properties and microstructure of cation intercalated Ti_3_C_2_T_x_ nanosheets, we took single cation intercalated D-Ti_3_C_2_T_x_-S and dual cations intercalated Alk-Ti_3_C_2_T_x_-S as parallel examples for comparison. From the curves in Figure 3A, the adsorption equilibrium of all samples was achieved within 15 min. The adsorption capacity of ternary cations-intercalated Alk-Ti_3_C_2_T_x_-M can reach as high as 183.5 mg/g, which was much higher than the reported Ti_3_C_2_T_x_-based nanomaterials. In particular, 93.9% of the equilibrium adsorption amount of lead ions was achieved in first 5 min for Alk-Ti_3_C_2_T_x_-M sample. The successive addition of LiF, KF, and NaOH through ternary cations intercalation in Alk-Ti_3_C_2_T_x_-M significantly broadened the interlayer spacing, thus promoting lead ions transporting more easily in interlayer voids. The adsorption kinetics of prepared D-Ti_3_C_2_T_x_-S, D-Ti_3_C_2_T_x_-M, Alk-Ti_3_C_2_T_x_-S, and Alk-Ti_3_C_2_T_x_-M product was fitted by pseudo second-order kinetic model (Figure S11). The regression coefficient value in second-order kinetic model for Alk-Ti_3_C_2_T_x_-M sample was 0.9999 (Table S3), which was higher than that of other samples. The effects of temperature and pH on the adsorption experiment for Alk-Ti_3_C_2_T_x_-M sample were studied to discover the optimal adsorption conditions and provide the guidance for the industrial adsorption application of MXenes. The adsorption capacity of Alk-Ti_3_C_2_T_x_-M was enhanced gradually with the increase of temperature. Among them, the equilibrium adsorption capacity of Alk-Ti_3_C_2_T_x_-M can reach 209.4 mg/g at 40°C with a pH of 6 (Figure S12). The results demonstrated that the adsorption of lead ions by Alk-Ti_3_C_2_T_x_-M was an endothermic process; the adsorption equilibrium shifts toward the positive reaction direction as the temperature rise, consequently achieving high adsorption capacity. On the other hand, when the solution was weak acidity or neutral, the adsorption of lead ions by ternary cations-intercalated Alk-Ti_3_C_2_T_x_-M was favorable.

The relationship between lead ions solution (C_e_) and equilibrium adsorption capacity (Q_e_) of Alk-Ti_3_C_2_T_x_-M was evaluated by Langmuir and Freundlich adsorption isotherms models (Figure 3B); the results showed the correlation coefficient of R^2^ was 0.93 and 0.80, respectively, indicating that the adsorption process of Alk-Ti_3_C_2_T_x_-M was more appropriately fitted by the Langmuir adsorption isotherms model. The Q_e_ of Alk-Ti_3_C_2_T_x_-M increased with the tendence of the concentration; the maximum capacity reached as high as ∼267.2 mg/g when the concentration of lead ions solution was 40 mg/L, which was very competitive compared with other adsorbents in reported literature (Figure 3C and Table S4). In particular, the prepared Alk-Ti_3_C_2_T_x_-M can cut down lead concentration from 10 mg/L to extremely low 0.009 mg/L, which was below the drinking water standards (0.015 mg/L) (Figure 3D). The zeta potential of ternary cations-intercalated Alk-Ti_3_C_2_T_x_-M nanosheets was negatively charged at a pH range of 3–10 (Figure 3E); the negative charge on the surface of MXene enhanced by increasing the pH of the media and the electrostatic attraction forces facilitated strong interactions between lead ions and Alk-Ti_3_C_2_T_x_ nanosheets, thus resulting in an increase of lead ions adsorbed on the surface of MXene. Importantly, the Alk-Ti_3_C_2_T_x_-M were more negative than Alk-Ti_3_C_2_T_x_-S, revealing more negatively charged functional groups existed on the surface of Alk-Ti_3_C_2_T_x_-M. However, the capacity decreased when the value of pH reach 8. The lead ions tended to form hydroxide complex precipitate in the alkaline environment; the generated hydroxide drops out from the solution and no free lead ions were adsorbed on the surface of Alk-Ti_3_C_2_T_x_-M. Even so, synthetized ternary cations intercalated Alk-Ti_3_C_2_T_x_-M can be readily regenerated and recycled for 5 cycles without significant loss of lead adsorption capacity (Figure S13), indicating great potential in removing heavy metal ions from water. The prepared ternary cations-intercalated MXene also exhibited high performance for other heavy ions; the adsorption capacities of Alk-Ti_3_C_2_T_x_-M toward Cr(Ⅵ), Cu(II), and Zn(II) were 188.5, 155.6, and 52.5 mg/g, respectively (Figure S14).

**Figure 4:**
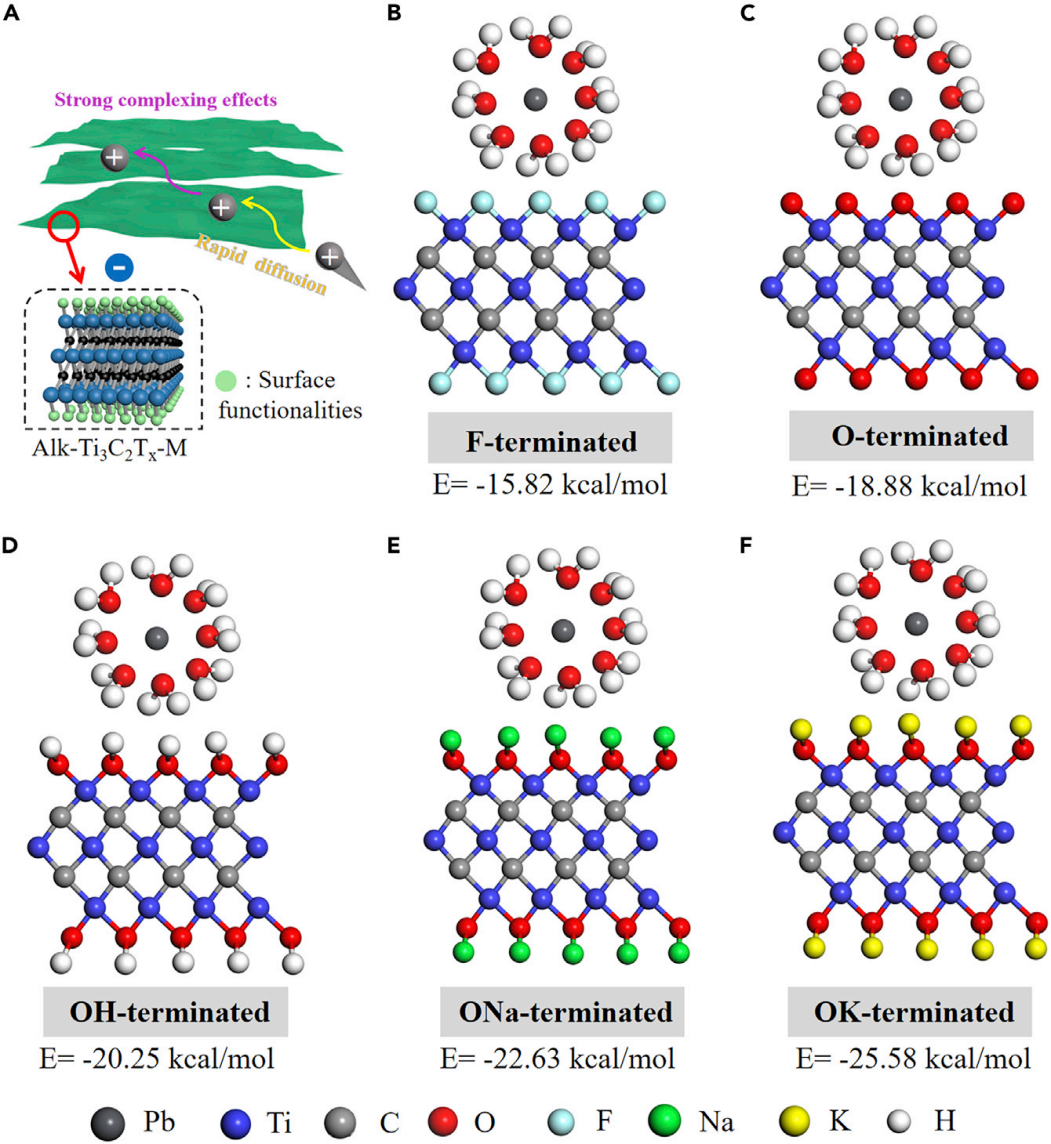
Molecular dynamics simulation (A) Schematic illustration of possible adsorption mechanism. (B–F) The electrostatic interaction energies between ternary cation-intercalated Ti_3_C_2_T_x_ with different functional groups and hydrated lead ion.

To verify the interactions between ternary cations-intercalated Ti_3_C_2_T_x_ MXene nanosheets and lead ions, the composition and surface chemical state of Alk-Ti_3_C_2_T_x_-M before and after adsorption test were evaluated. After the adsorption experiment of Alk-MXenes, a large amount of lead was found on the surface, and the content of Na and K on the surface was greatly reduced (Figure S15). This is because lead ions occupy the sites of -ONa and -OK. Figure 3F showed the FTIR diagram of the Alk-Ti_3_C_2_T_x_-M before and after adsorption. The shift of the -OH absorption peak (∼3448 cm^−1^), the characteristic absorption peak of the Ti-O deformation vibration peak is split into two peaks (555 and 520 cm^−1^),^12^ indicating that lead ions have a strong affinity with the functional group at the Ti site. By optimizing the microstructure and surface chemical state, high adsorption performance of ternary cations-intercalated Ti_3_C_2_T_x_ MXene can be achieved (Figure 4A). The main are as follows: (1) The restacking of Ti_3_C_2_T_x_ MXene nanosheets was significantly mitigated after intercalation of ternary cations, resulting in lead ions rapidly transporting in interlayer voids. (2) High surface area was achieved by precisely controlling the interlayer spacing at Å-size to generate abundant active sites for adsorption. (3) Surface functionalization improves the hydrophilicity of Ti_3_C_2_T_x_ intercalated with ternary cations to facilitate water flow (Figure S16). (4) Large numbers of fluorine groups were superseded by -OH, -ONa, and -OK groups to improve surface chemical state, thus promoting strong complexing effects to remove lead ions. We also carried molecular dynamic simulation to investigate the interactions among different functional groups and hydrated Pb^2+^ by employing the Materials Studio software package. The electrostatic interaction energies of Ti-OH-Pb (−20.25 kcal/mol), Ti-ONa-Pb (−22.63 kcal/mol), and Ti-OK-Pb (−25.58 kcal/mol) were stronger than that of Ti-F-Pb (−15.82 kcal/mol) and Ti-*O*-Pb (-18.88 kcal/mol) in established models (Figures 4B–4F), which demonstrated that surface Ti-OH, Ti-ONa, and Ti-OK groups possessed stronger metal-ligand interaction with hydrated lead ions. After the alkalization, plentiful F sites were substituted by OH, ONa, and OK groups, consequently strengthening the adsorption role of Alk-Ti_3_C_2_T_x_-M nanosheets.

### Conclusion

Ternary cations-intercalated Ti_3_C_2_T_x_ MXene nanosheets were synthesized by mixed fluoride salts wet etching and alkalization process. Lithium, potassium, and sodium ions were successively inserted into multilayered Ti_3_C_2_T_x_ to enlarge the distance between layers and selectively weaken the interaction forces, thus achieving structural control with angstrom-level precision and high surface area (92.6 m^2^ g^−1^). On account of high surface area which provided numerous active sites and abundant functional groups which displayed strong complexing effects with heavy metal ions, ternary cations-intercalated Ti_3_C_2_T_x_ MXene nanosheets exhibited a high adsorption capacity of 267.2 mg/g toward lead ions with lead concentration sharply cut down from 10 mg/L to extremely low 0.009 mg/L, which was far below the drinking water standards (0.015 mg/L). The yield of generated dispersed Ti_3_C_2_T_x_ can increase from 45% to 62.9% compared with reported method by single Li ion intercalation. Considering common Ti_3_C_2_T_x_ MXene nanosheets etched by single fluorinated salt are typically facing difficulties such as stacking of nanosheets and narrow interlayer distance, the strategy of constructing Ti_3_C_2_T_x_ MXene nanosheets with precisely controlled microstructure can be used for many other applications including supercapacitors, membranes, and sensors.

### Limitations of the study

Until now, it is very hard to quantitatively investigate the interactions between targets and adsorbents by characterizations. Although theoretical models were established to qualitatively describe the adsorption behavior, more exquisite theoretical models are necessary for quantitatively illustrating the interfacial interactions during adsorption process.

## STAR★Methods

### Key resources table

**Table:** 

REAGENT or RESOURCE	SOURCE	IDENTIFIER
**Chemicals and materials**		

Ternary layered ceramics Ti_3_AlC_2_ (>99.0%)	Beijing Advanced 2D Materials	CAS: 196,506-01-1
Sodium hydroxide (>97.0 %)	Aladdin	CAS: 1310-73-2
Ethyl alcohol (>99.0 %)	Aladdin	CAS: 64-17-5
Potassium fluoride (>99.0 %)	Aladdin	CAS: 7789-23-3
Lithium fluoride (>99.0 %)	Aladdin	CAS: 7789-24-4
Hydrochloric acid (37.0 %)	Shanghai Sinopharm Chemical	CAS: 7647-01-0
Lead nitrate (>99.0 %)	Aladdin	CAS: 10099-74-8
Cr(Ⅵ) (1000 μg/mL)	Aladdin	CAS: 7440-47-3
Copper nitrate (>99.0%)	Aladdin	CAS: 10,031-43-3
Zinc nitrate (>99.0%)	Aladdin	CAS: 10,196-18-6
Argon gas (>98.0%)	Hangzhou Jinggong Special Gas	CAS: 7440-37-1

**Software and algorithms**		

Materials Studio software	Accelrys Incorporation	N/A
Nano measurer software	Analytical Software Website	https://www.jb51.net/softs/583257.html

### Resource availability

#### Lead contact

Further information and requests for resources should be directed to the lead contact, Guoliang Zhang (guoliangz@zjut.edu.cn).

#### Materials availability

This work did not generate new unique reagents.

### Experimental model and subject details

Any animals, human subjects, plants, microbe strains, cell lines, primary cell cultures were not used in the study.

### Method details

#### Preparation of multilayered Ti_3_C_2_T_x_-M

A certain amount of LiF was dissolved into 20 mL HCl solution. Then, Ti_3_AlC_2_ powders were slowly added to the corrosive agent above 10 min. After that, the device was sealed and argon gas was selected as protective gas. Next, the mixed solution was stirred at different temperature for 24 h. Subsequently, KF was added into the solution and stirred for another 24 h. After the upper suspension was removed, the mixture was centrifuged at 3500 rpm for 5 min. And the sediment was washed for several times with deionized water, and then washed twice with ethanol. At last, the sediment was collected and dried overnight at 60°C in vacuum for obtaining multilayered Ti_3_C_2_T_x_ (labeled as Ti_3_C_2_T_x_-M), and it was stored in a low-temperature and dry environment. The Ti_3_C_2_T_x_-S sample was prepared in a similar way by using single fluorinated salt (LiF).

#### Preparation of D-Ti_3_C_2_T_x_-M and Alk-Ti_3_C_2_T_x_-M

0.2 g multilayered Ti_3_C_2_T_x_-M was put into the distilled water with a mass ratio of 1:300. After ultrasonic treatment for 1 h under argon atomosphere, the sample was centrifuged at 3500 rpm for 1 h to obtain the suspension of delaminated Ti_3_C_2_T_x_-M (labeled as D-Ti_3_C_2_T_x_-M). Vacuum filtration was used to calculated of the quality of D-Ti_3_C_2_T_x_-M in suspension. After drying at 60°C in vacuum condition for 12 h, the total mass of the D-Ti_3_C_2_T_x_-M and the membrane was weighed, thus calculating the concentration and quality of D-Ti_3_C_2_T_x_-M in the suspension and the exfoliating efficiency by related formula. The D-Ti_3_C_2_T_x_-M was further placed in a 4 wt % sodium hydroxide solution and stirred at room temperature for 2 h for alkalinization experiments. After the reaction, the samples were centrifuged at 8000 rpm for 5 min, then washed with deionized water and ethanol for several times, respectively. The sediment was collected and vacuum dried at 60°C for 12 h to obtain alkalized D-Ti_3_C_2_T_x_-M nanosheets (labeled as Alk-Ti_3_C_2_T_x_-M). The D-Ti_3_C_2_T_x_-S and Alk-Ti_3_C_2_T_x_-S nanosheets were synthesized in a similar way.

#### Adsorption experiments

The adsorption performance of prepared samples were tested in a stock of lead ions solution (20 mg/L). For adsorption studies, 0.01 g sample was added to lead ions solution in a shaking incubator, the mixed solution was measured with a needle filter after different time intervals. Then, the concentration of lead ions in the solution was measured by atomic absorption spectrometry and the content of residual lead ions was calculated. In addition, the effects of pH and temperature on the adsorption performance of Alk-Ti_3_C_2_T_x_-M were also investigated. The pH values of the lead ions solution were adjusted to 4–8 and the temperature was set at 20–40°C.

#### Characterizations

The surface morphology and elemental analysis of prepared MXenes under different experimental conditions were evaluated by an SU8010 scanning electron microscope (SEM) (Hitachi, Japan) at 15 kV and corresponding energy dispersive X-ray spectroscope (EDX), respectively. Microstructural characterizations of D-Ti_3_C_2_T_x_-S, D-Ti_3_C_2_T_x_-M, and Alk-Ti_3_C_2_T_x_-M nanosheets were observed by a Tecnai G2 F30 S-Twin transmission electron microscope (TEM) (Philips-FEI, Netherlands). The thickness of D-Ti_3_C_2_T_x_-M, and Alk-Ti_3_C_2_T_x_-M nanosheets were detected by atomic force microscope (AFM, Dimension Icon). Nicolet 6700 Fourier transform infrared (FTIR) (Thermo Nicolet, America) spectra with the wavenumber range of 450–4000 cm^−1^, which was used for analyzing the surface functional groups of prepared MXenes. The surface chemical states of synthesized MXenes were further verified by X-ray photoelectron spectroscopy (XPS) (PHI-5000C, America). The crystalline structure of synthesized MXenes were performed by a X’Pert PRO X-ray powder diffraction (XRD) (PNAlytical, Netherlands) with a scan step of 0.015° in the scan region of 5–55°. The Brunauer-Emmet-Teller (BET) surface area was analyzed by an ASAP 2020 analyzer (Micromeritics, USA). The atomic absorption spectrometer (Varian, USA) was used to measure the concentration of lead ions in the solution. The zeta potential of synthesized MXene samples were analyzed by using KCl aqueous solution.

#### Molecular dynamics simulations

The molecular dynamics simulations were employed to obtain the electrostatic interaction energies by using the Materials Studio software package. The time step was set as 1.0 fs and the COMPASS force field was used with the Berendsen algorithm to maintain a constant temperature (298 K). The atomistic structures were optimized by energy minimization method and then 200 ps NVE-MD runs were used to further equilibrate the models. The electrostatic interaction energy between functional groups in Alk-Ti_3_C_2_T_x_-M and hydrated lead ion was defined as:

$$E=E_{functional\;groups+hydrated\;lead\;ion}-\left(E_{functional\;groups}+E_{hydrated\;lead\;ion}\right)$$

Where E_functional groups+hydrated lead ion_ is the total energy of functional groups in Alk-Ti_3_C_2_T_x_-M and hydrated lead ion; E_functional groups_ and E_hydrated_ _lead ion_ are total energy of functional groups in Alk-Ti_3_C_2_T_x_-M and hydrated lead ion, respectively.

### Quantification and statistical analysis

The thickness of MXene nanosheets was evaluated by Nano Measurer software, and error bars represent standard deviations for at least 5 measurements. Data are presented as mean ±. Moreover, the interlayer spacing in different samples was obtained by Nano Measurer software. Data are presented as mean ± HRTEM.

### Additional resources

There is no additional resources need to be declared in this manuscript, additional requests for this can be made by contacting the lead contact.

## Data Availability

• Data reported in this article will be shared by the lead contact on request. • There is no dataset or code associated with this work. • Any additional information required to reanalyse the data reported in this study is available from the lead contact upon request.

## Supplemental information

Document S1.Figures S1−S16 and Tables S1−S4
