# Supplementary material for: Two-dimensional titanium carbide MXene produced by ternary cations intercalation via structural control with angstrom-level precision
Source: iScience. 2022 Nov 15;25(12):105562. doi: 10.1016/j.isci.2022.105562 (PMC9703608; doi:10.1016/j.isci.2022.105562)
Supplement: Document S1.Figures S1−S16 and Tables S1−S4 [file mmc1.pdf]

iScience, Volume *25*

Supplemental information

Two-dimensional titanium carbide MXene produced by ternary cations intercalation via structural control with angstrom-level precision

Zehai Xu, Yufan Zhang, Minmin Liu, Qin Meng, Chong Shen, Lushen Xu, Guoliang Zhang, and Congjie Gao

Supplementary Information

Two-dimensional titanium carbide ($Ti_3C_2T_x$) MXene produced by ternary cations intercalation via structural control with angstrom-level precision

Zehai Xu [1,4], Yufan Zhang [2,4], Minmin Liu [1,4], Qin Meng [3], Chong Shen [1,3], Lushen Xu [1], Guoliang Zhang [1,5,*], Congjie Gao [1]

[1] Center for Membrane and Water Science & Technology, Institute of Oceanic and Environmental Chemical Engineering, State Key Lab Breeding Base of Green Chemical Synthesis Technology, Zhejiang University of Technology, Hangzhou 310014, P. R. China.

[2] College of Engineering, Carnegie Mellon University, Pittsburgh, PA 15213, USA.

[3] College of Chemical and Biological Engineering, State Key Laboratory of Chemical Engineering, Zhejiang University, Hangzhou 310027, P. R. China.

[4] These authors contributed equally to this work.

[5] Lead Contact.

[*] Correspondence to: guoliangz@zjut.edu.cn (G.Z.)

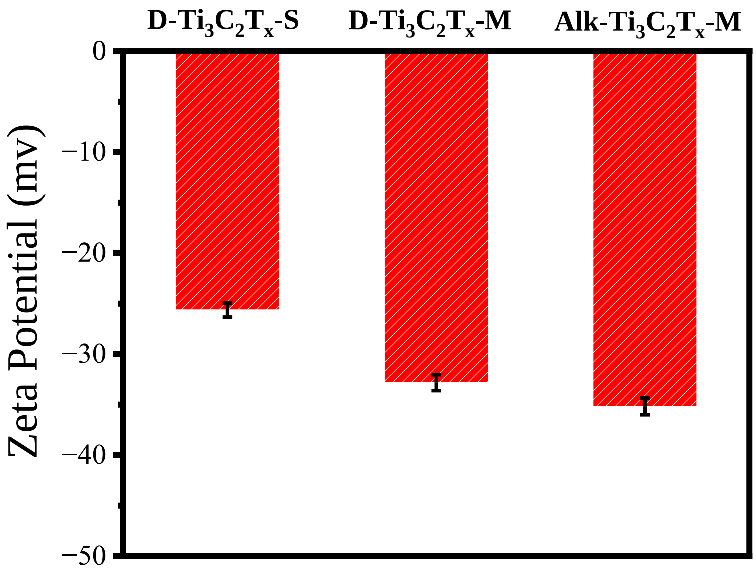

Figure S1 Zeta potential of D-$Ti_3C_2T_x$-S, D-$Ti_3C_2T_x$-M and Alk-$Ti_3C_2T_x$-M (pH=7). Related to Figure 1.

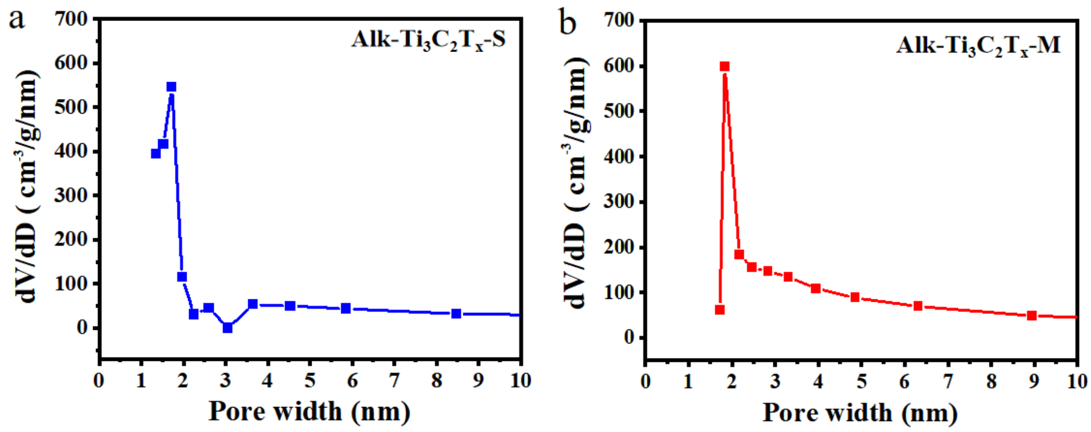

Figure S2 Pore size distribution for Alk-$Ti_3C_2T_x$-S (a) and Alk-$Ti_3C_2T_x$-M (b).

Related to Figure 1.

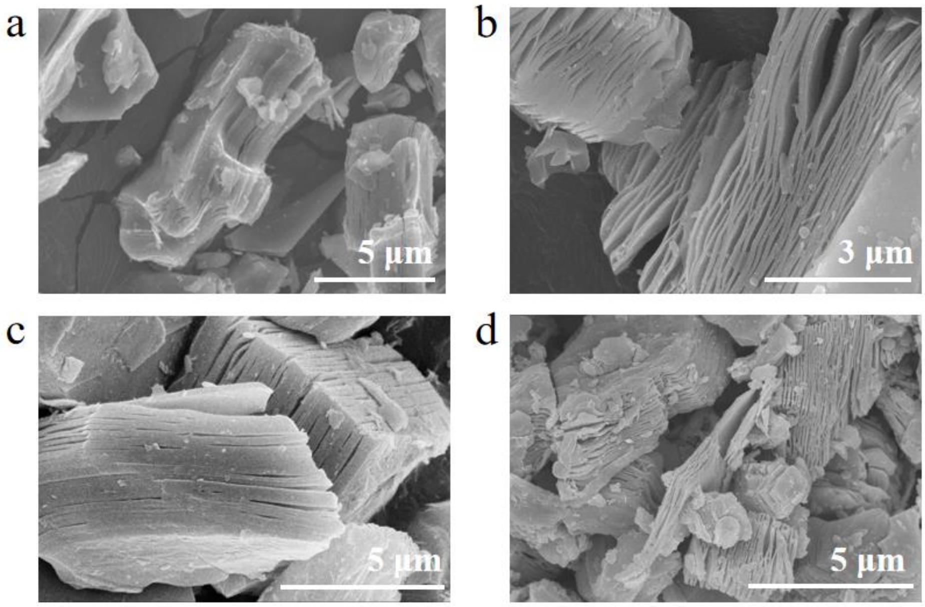

Figure S3 Morphology of mutilayered $Ti_3C_2T_x$-M synthesized under different conditions. SEM images of mutilayered $Ti_3C_2T_x$-M synthesized with a molar ratio of LiF:KF=3:1 (**a**) and 7:1 (**b**). SEM images of mutilayered $Ti_3C_2T_x$-M prepared under etching temperature of 30 ℃ (**c**) and 40 ℃ (**d**). Related to Figure 2.

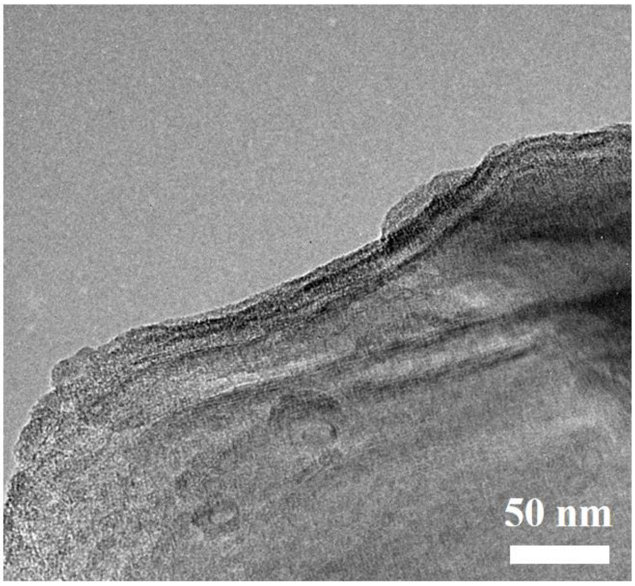

Figure S4 TEM image of D-$Ti_3C_2T_x$-S sample. Related to Figure 2.

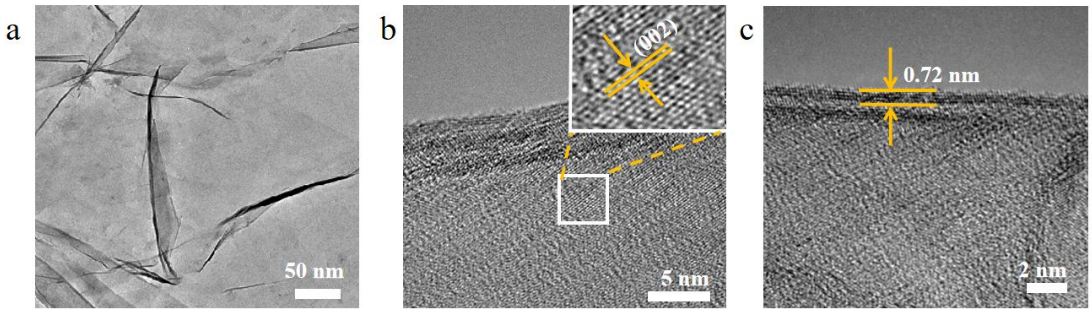

Figure S5 TEM and HRTEM images. (**i**) TEM image of D-$Ti_3C_2T_x$-M. (**j**, **k**) HRTEM images of D-$Ti_3C_2T_x$-M. The inset in (**j**) showing the lattice of synthesized D-$Ti_3C_2T_x$-M. Related to Figure 2.

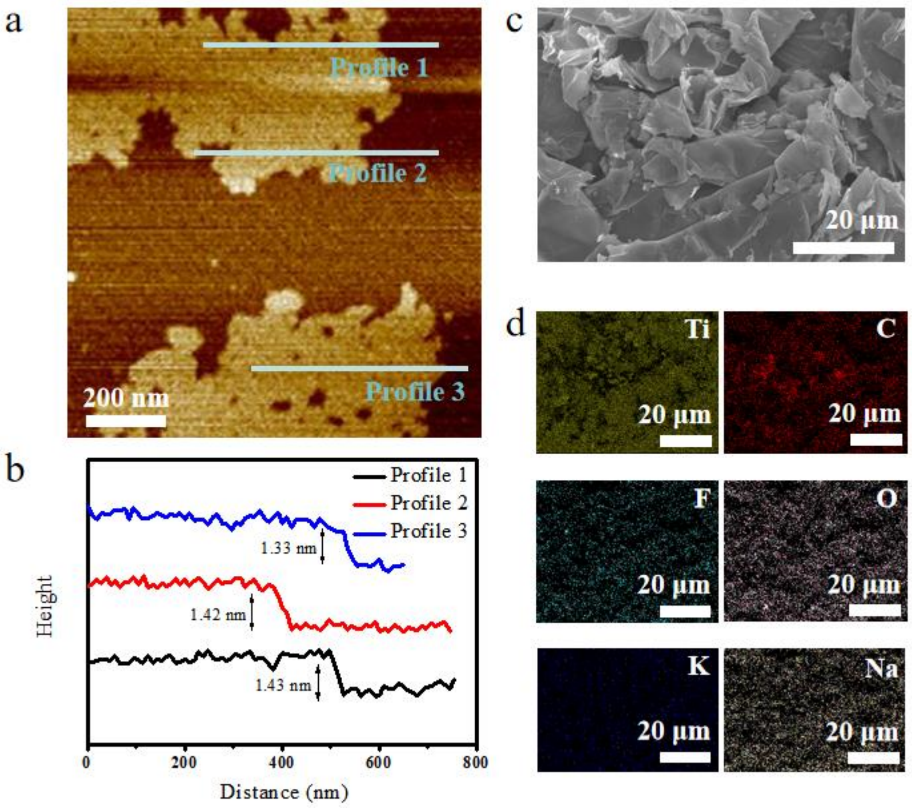

Figure S6 Morphology and composition of $Ti_3C_2T_x$ MXene nanosheets by ternary cation intercalation method. (a, b) AFM image and corresponding height profiles of Alk-$Ti_3C_2T_x$-M nanosheets. (**c**, **d**) SEM image of Alk-$Ti_3C_2T_x$-M and corresponding element mapping. Related to Figure 2.

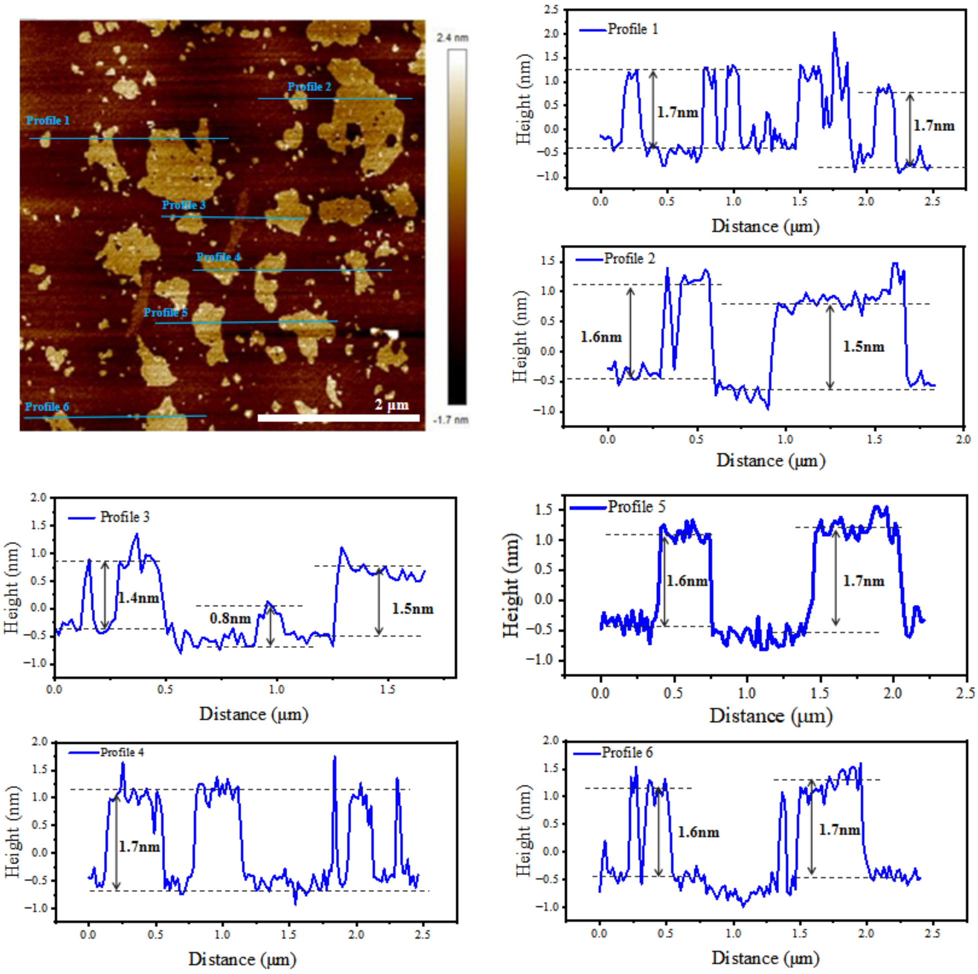

Figure S7 AFM image and corresponding height profiles of D-$Ti_3C_2T_x$-M. Related to Figure 2.

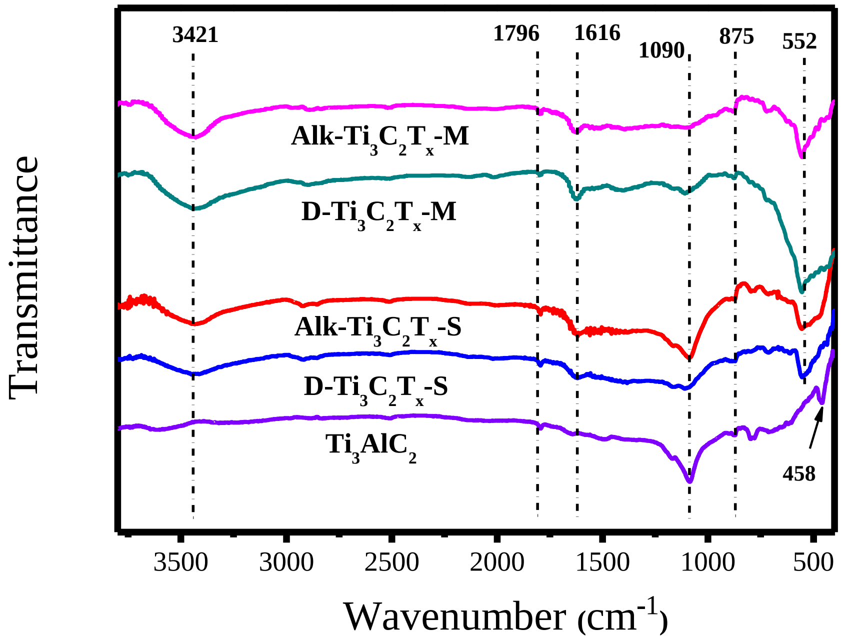

Figure S8 FTIR spectra of prepared samples. The absorption peaks at 1090 cm^{-1}, 875 cm^{-1} and 713 cm^{-1} in Ti_3AlC_2 correspond to the stretching vibration peak and bending vibration peak of C-O, respectively. The characteristic absorption peak of $Ti_3C_2T_x$ appears at around 552 cm^{-1}, the deformation vibration peak of Ti-O revealed that an oxygen-containing functional group appeared on the Ti site of $Ti_3C_2T_x$ to form Ti-OH or Ti-O-Ti bonds. The infrared absorption peak at 458 cm^{-1} was attributed to the deformation C-O-C bond. Related to Figure 3.

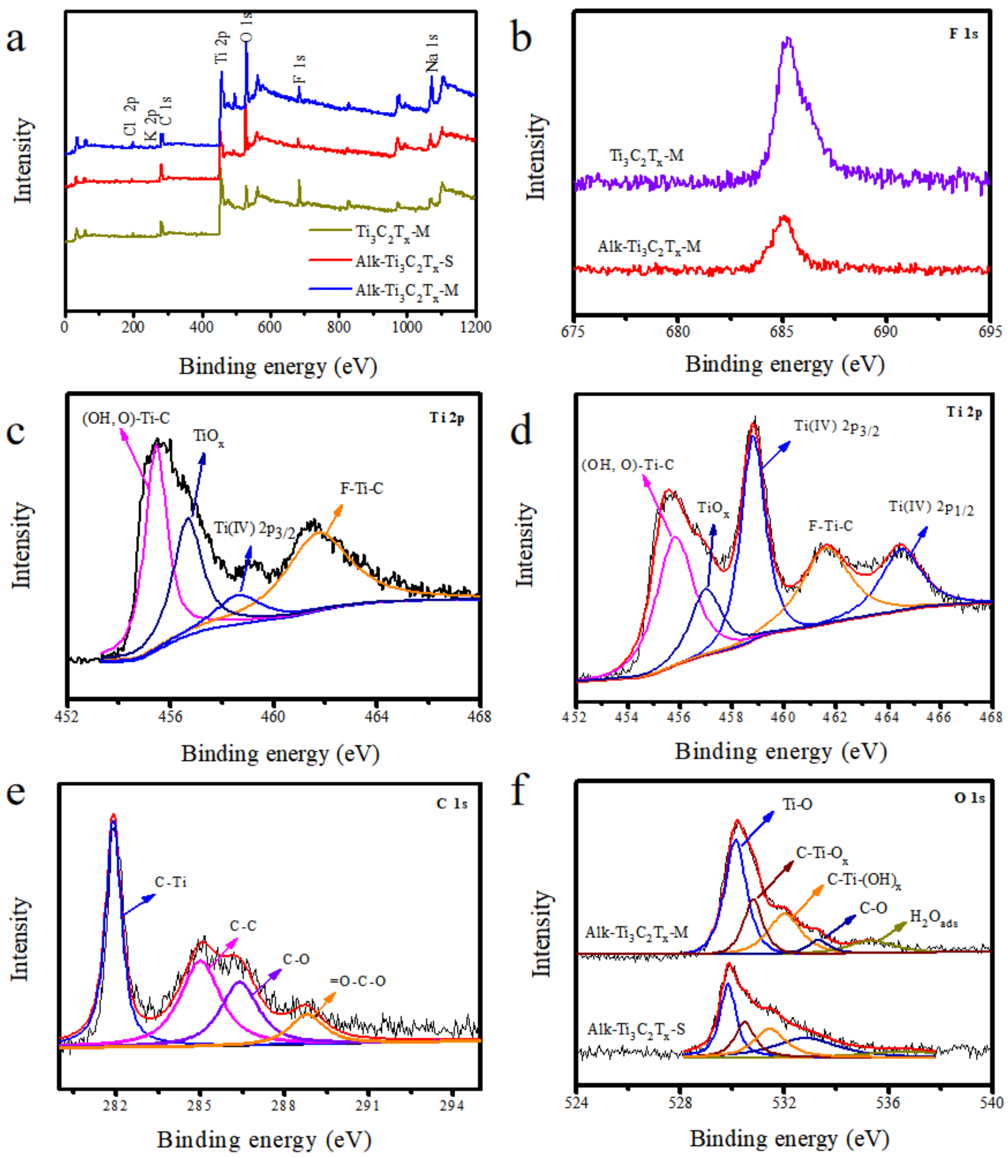

Figure S9 XPS spectra. (**a**) full spectra. (**b**) F 1s spectra of D-$Ti_3C_2T_x$-M and Alk-$Ti_3C_2T_x$-M. Ti 2p spectra of D-$Ti_3C_2T_x$-M (**c**) and Alk-$Ti_3C_2T_x$-M (**d**). (**e**) C 1s spectra of Alk-$Ti_3C_2T_x$-M. (**f**) O 1s spectra of Alk-$Ti_3C_2T_x$-S and Alk-$Ti_3C_2T_x$-M. The Ti 2p spectra of Alk-$Ti_3C_2T_x$-M can be resolved into five peaks: (OH, O)-Ti-C (455.8 eV), TiO_x (457.2 eV), Ti(IV) $2p_{3/2}$ (458.8 eV), Ti-F (461.9 eV) and Ti (IV) $2p_{1/2}$ (464.7 eV). Related to Figure 3.

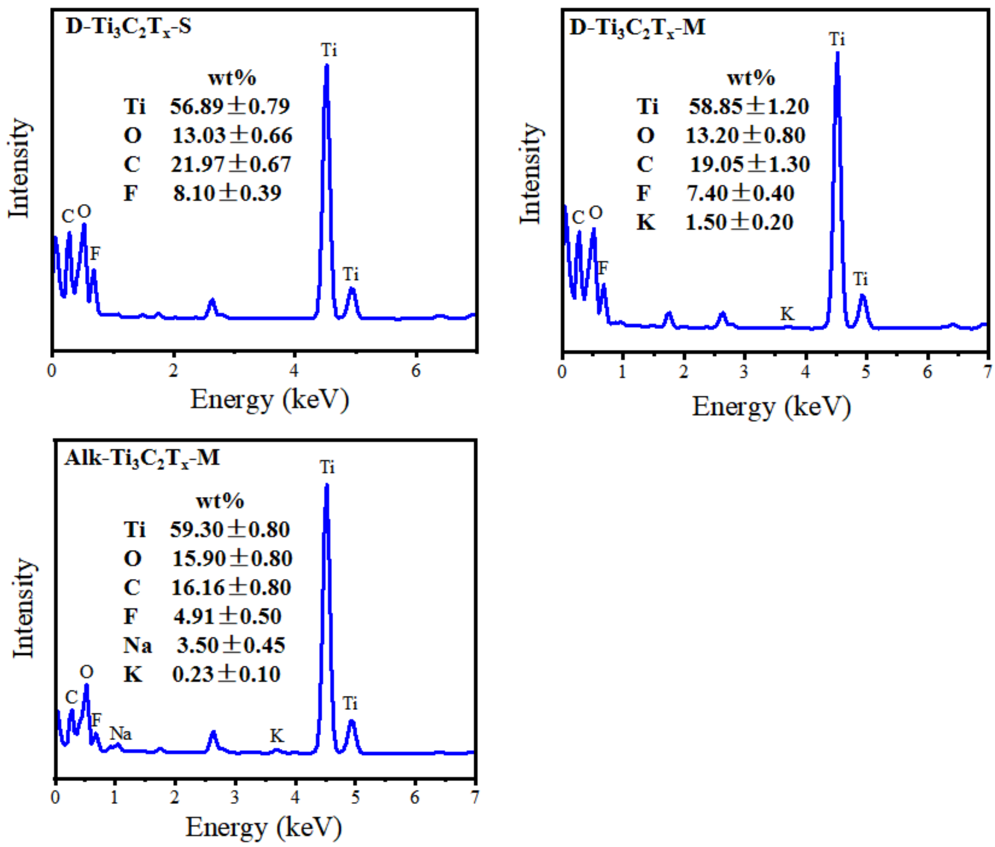

Figure S10 EDX analysis of D-$Ti_3C_2T_x$-S, D-$Ti_3C_2T_x$-M and Alk-$Ti_3C_2T_x$-M.

Related to Figure 3.

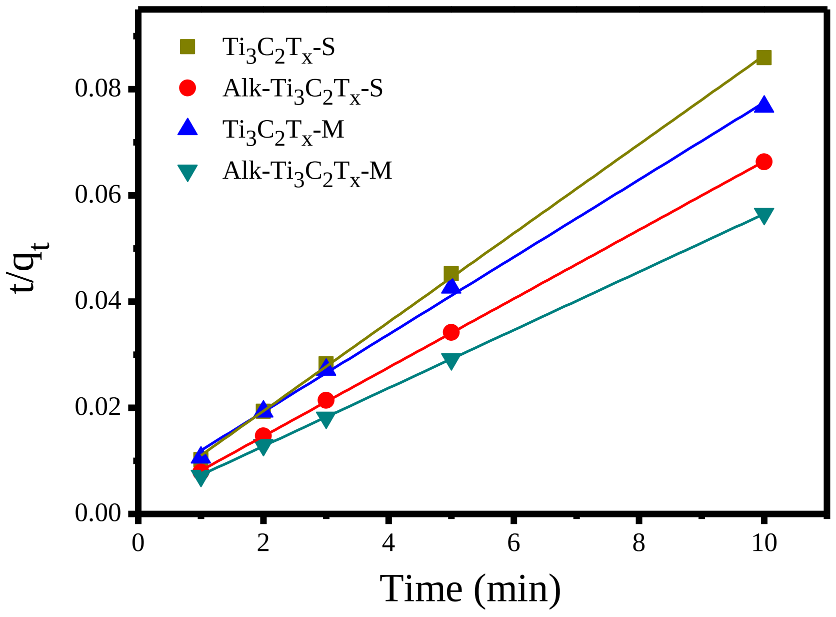

Figure S11 Pseudo-second-order kinetic graph. Related to Figure 3.

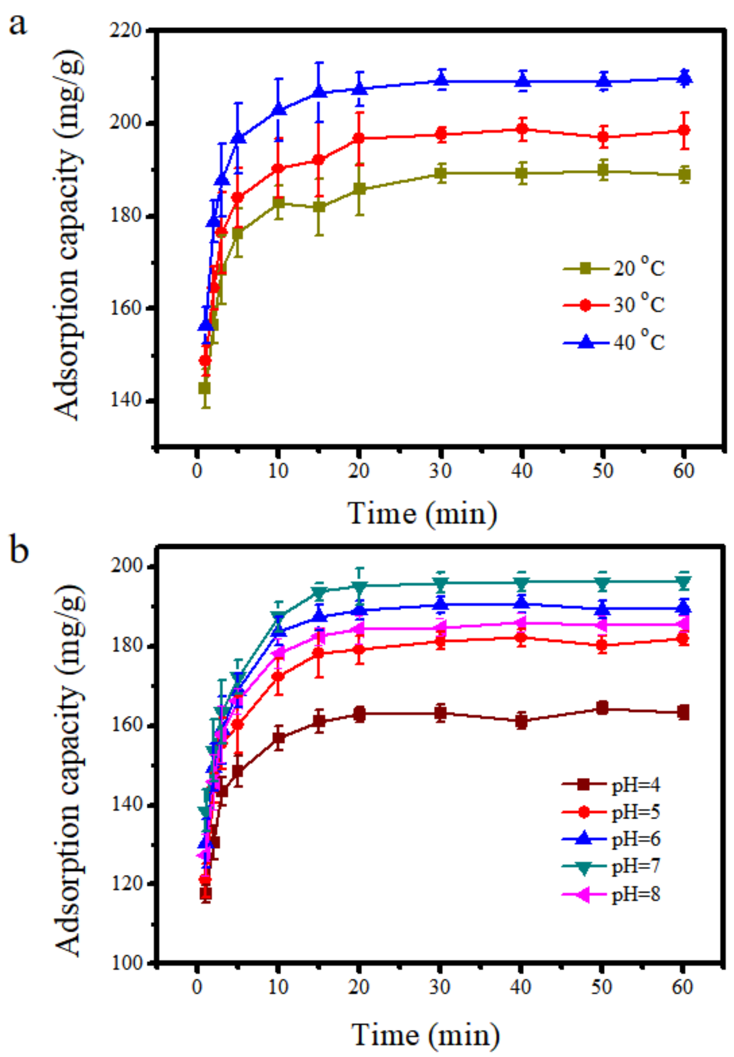

Figure S12 The effect of temperature (a) and pH (b) on adsorption performance of Alk-$Ti_3C_2T_x$-M. Related to Figure 3.

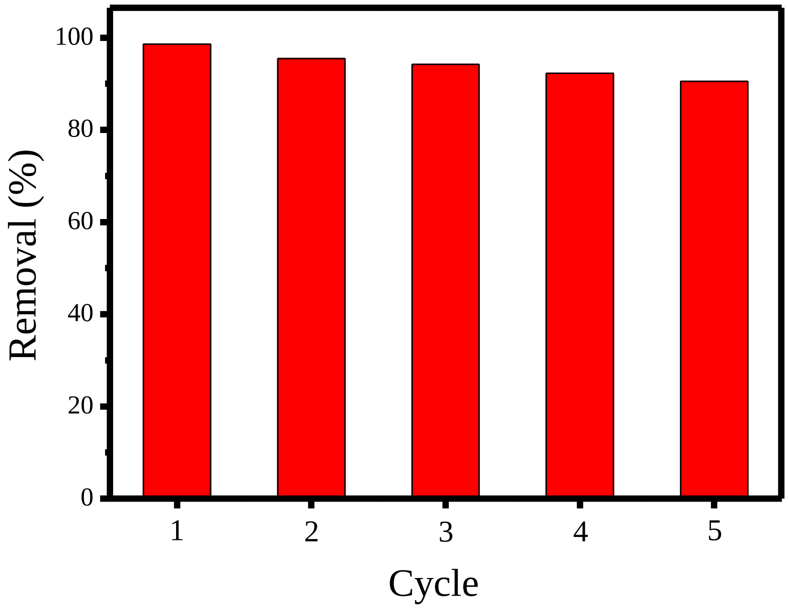

Figure S13 Pb(II) adsorption capability by regenerated Alk-$Ti_3C_2T_x$-M for 5 cycles. Related to Figure 3.

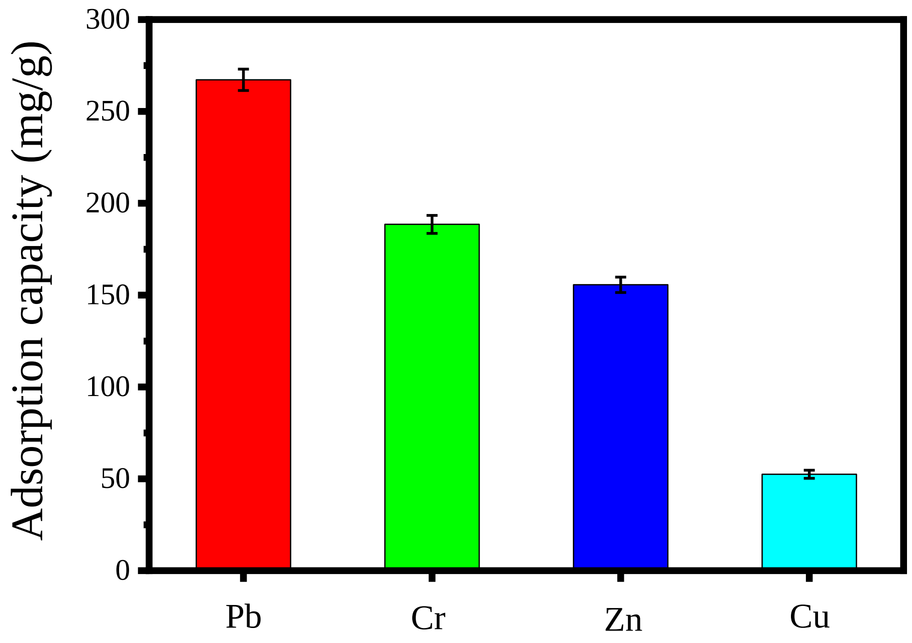

Figure S14 Removal capacity test of Alk-$Ti_3C_2T_x$-M toward Pb(II), Cr(VI), Cu(II) and Zn(II). Related to Figure 3.

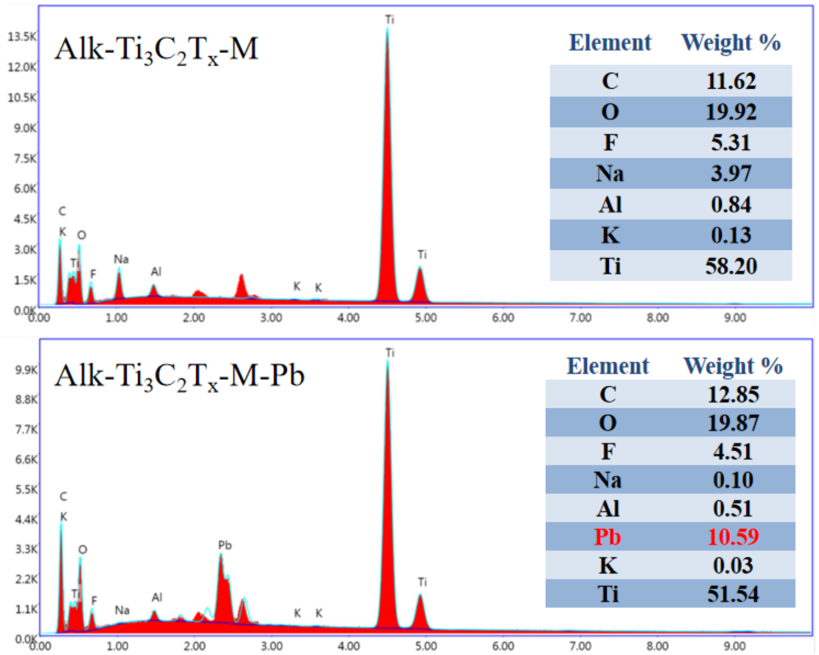

Figure S15 EDX analysis of Alk-$Ti_3C_2T_x$-M sample before and after adsorption of lead ions. Related to Figure 4.

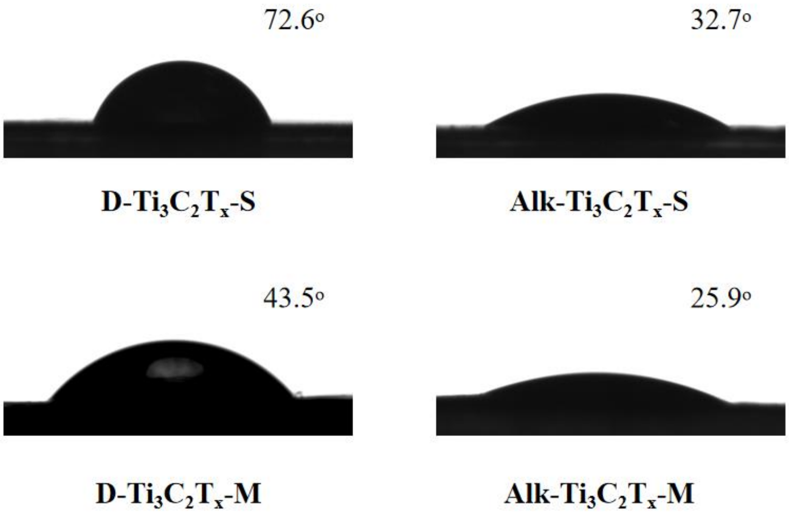

Figure S16 Contact angle of synthesized MXene. Related to Figure 4.

Table S1 Elemental composition of different samples

Sample	Content (at. %)						
	Ti	C	Al	O	F	K	Na
Ti_3AlC_2	60.64	19.29	8.27	11.80	/	/	/
D-$Ti_3C_2T_x$-M	58.27	13.28	0.54	17.17	10.53	0.21	/
Alk-$Ti_3C_2T_x$-M	58.20	11.62	0.84	19.92	5.31	0.13	3.97

Related to Figure 2.

Table S2 The yield of D-$Ti_3C_2T_x$-M under different experimental conditions.

Conditions		Yield (%)
30 ℃	LiF:KF=5:1	18.2
35 ℃	LiF	43.7
	LiF:KF=3:1	38.9
	LiF:KF=5:1	62.9
	LiF:KF=7:1	52.7
40 ℃	LiF:KF=5:1	60.2

Related to Figure 2.

Table S3 The fitting parameterso f adsorption kinetic models.

Sample	pseudo second order kinetic	
	k_1 ×10^{-3} (g/mg ·min)	R^2
D-$Ti_3C_2T_x$-S	8.37	0.9974
Alk-$Ti_3C_2T_x$-S	6.47	0.9998
D-$Ti_3C_2T_x$-M	7.28	0.9993
Alk-$Ti_3C_2T_x$-M	5.47	0.9999

Related to Figure 3.

Table S4 Comparison about adsorption performance of lead ions on various adsorbents.

Sample	Condition	Equilibrium time (min）	Adsorption capacity (mg/g)	Ref
Activated carbon	pH=6; T=303 K	60	21.80	S1
Na-bentonite	pH=4; T=298 K	~150	47.8	S2
TiO_2/CF	pH=6; T=298 K	240	11.3	S3
TiO_2-SiO_2/lignin	T=293 K	30	59.9	S4
TiO_2/cellulose	pH=6; T=298 K	30	42.5	S5
Salecan-g-PAS Hydrogel	pH=7.4; T=298 K	100	172.8	S6
Fe_3O_4@SiO_2@CS-P	pH=6; T=298 K	90min	151.8 mg/g	S7
Amide-based COFs	pH=4.5	25	185.7	S8
CNTs/Fe_3O_4-NH_2	pH=5.3; T=303 K	/	75.0	S9
SiO_2@MoS_2	pH=6	/	199	S10
$K_2W_4O_{13}$	pH=5.3; T=298 K	180	228.83	S11
$Ti_3C_2T_x$-KH570	pH=4-4.5; T=303 K	30	147.29	S12
NH_2-M-AGS	pH=5.5; T=298 K	400	127.7	S13
Alk-$Ti_3C_2T_x$	pH=6.5; T=323 K	2	~140	12
$Ti_3C_2T_x$ MXene	pH=6.0; T=293 K	30	~35	S14
EHL-$Ti_3C_2T_X$	pH=5.0; T=303 K	300	239	S15
1T-MoS_2	pH=5.0; T=298 K	20	147.09	S16
g-C_3N_4/MnO_2	pH=5.0; T=298 K	270	204.1	S17
Zn(Bim)(OAc)	pH=6.0; T=298 K	180	253	S18
O-TMU-40	pH=7.0; T=298 K	120	215	S19
sulfur-doped -C_3N_4	pH=4.5; T=298 K	120	52.63	S20
Fe_3O_4/poly($C_3N_3S_3$)	pH=6.0; T=298 K	20	232.6	S21
Alk-$Ti_3C_2T_x$-M	pH=6; T=313 K	15<br>First 2 min	267.2<br>227.5	This work

Related to Figure 3.

Supplementary references

S1. Rao, M. M., Ramana, D. K., Seshaiah, K., Wang, M. C., Chang Chien, S. W. (2009). Removal of some metal ions by activated carbon prepared from Phaseolus aureus hulls. J. Hazard. Mater. *166*, 1006-1013. https://doi.org/10.1016/j.jhazmat.2008.12.002.

S2. Yang, S. T., Zhao, D. L., Zhang, H., Luo, F. (2010). Impact of environmental conditions on the sorption behavior of Pb (II) in Na-bentonite suspensions. J. Hazard. Mater. *183*, 632-640. https://doi.org/10.1016/j.jhazmat.2010.07.072.

S3. Li, Y., Li, L., Cao, L., Yang, C. (2016). Promoting dynamic adsorption of Pb^{2+} in a single pass flow using fibrous nano-TiO_2/cellulose membranes. Chem. Eng. J. *283*, 1145-1153. https://doi.org/10.1016/j.cej.2015.08.068.

S4. Klapiszewski, Ł., Siwińska-Stefańska, K., Kołodyńska, D. (2017). Preparation and characterization of novel TiO_2/lignin and TiO_2-SiO_2/lignin hybrids and their use as functional biosorbents for Pb (II). Chem. Eng. J. *314*, 169-18. https://doi.org/10.1016/j.cej.2016.12.114.

S5. Zhang, J., Li, L., Li, Y., Yang, C. (2017). Microwave-assisted synthesis of hierarchical mesoporous nano-TiO_2/cellulose composites for rapid adsorption of Pb^{2+}. Chem. Eng. J. *313*, 1132-1141. https://doi.org/10.1016/j.cej.2016.11.007.

S6. Qi, X. L., Lin, L., Shen, L. L., Li, Z. P., Qin, T., Qian, Y. N., Wu, X., Wei, X., Gong, Q. W., Shen, J. L. (2019). Efficient decontamination of lead ions from wastewater by salecan polysaccharide-based hydrogels. ACS. Sustain. Chem. Eng. *7*, 11014-11023. https://doi.org/10.1021/acssuschemeng.9b02139.

S7. Huang, Y., Hu, C., An, Y., Xiong, Z., Hu, X., Zhang, G., Zheng, H. (2021). Magnetic phosphorylated chitosan composite as a novel adsorbent for highly effective and selective capture of lead from aqueous solution. J. Hazard. Mater. *405*, 124195.https://doi.org/10.1016/j.jhazmat.2020.124195

S8. Li, G. L., Ye, J. R., Fang, Q. L., Fu, L. (2019). Amide-based covalent organic frameworks materials for efficient and recyclable removal of heavy metal lead (II) Chem. Eng. J. *370*, 822-830. https://doi.org/10.1016/j.cej.2019.03.260.

S9. Ji, L., Zhou, L., Bai, X., Shao, Y., Zhao, G., Qu, Y., Wang, C., Li, Y. (2012). Facile synthesis of multiwall carbon nanotubes/iron oxides for removal of tetrabromobisphenol A and Pb (II) J. Mater. Chem. *22*, 15853-15862. DOI10.1039/c2jm32896h.

S10. Mondal, B., Mahendranath, A., Som, A., Bose, S., Ahuja, T., Kumar, A. A., Ghosh, J., Pradeep, T. (2018). Rapid reaction of MoS_2 nanosheets with Pb^{2+} and Pb^{4+} ions in solution. Nanoscale *10*, 1807-1814. https://doi.org/10.1039/c7nr07523e.

S11. Huang, Q. S., Wu, W., Wei, W., Song, L., Sun, J., Ni, B.J. (2020). Highly-efficient Pb^{2+} removal from water by novel $K_2W_4O_{13}$ nanowires: Performance, mechanisms and DFT calculation. Chem. Eng. J. *381***,** 469-477. https://doi.org/10.1016/j.cej.2019.122632.

S12. Du, Y. C., Yu, B., Wei, L.Q., Wang, Y. L., Zhang, X. M., Ye, S.F. (2019). Efficient removal of Pb(II) by $Ti_3C_2T_x$ powder modified with a silane coupling agent. J. Mater. Sci. *54*, 13283–13297. https://doi.org/10.1007/s10853-019-03814-z.

S13. Huang, X., Wei, D., Zhang, X. W., Fan, D. W., Sun, X. Du, B., Wei, Q. (2019). Synthesis of amino-functionalized magnetic aerobic granular sludge-biochar for Pb(II) removal: Adsorption performance and mechanism studies. Sci. Total. Environ. *685*, 681-689. https://doi.org/10.1016/j.scitotenv.2019.05.429.

S14. Jun, B. M., Her, N., Park, C. M., Yoon, Y. (2020). Effective removal of PbIJII) from synthetic wastewater using $Ti_3C_2T_x$ MXene. Environ. Sci. Water Res. Technol. *6*, 173-180. https://doi.org/10.1039/c9ew00625g.

S15. Wang, S. H., Liu, Y. L., Lü, Q. F., Zhuang, H. P. (2020). Facile preparation of biosurfactant-functionalized Ti_2CT_X MXene nanosheets with an enhanced adsorption performance for Pb(II) ions. J. Mol. Liq. *297*, 111810. https://doi.org/10.1016/j.molliq.2019.111810.

S16. Luo, J. M., Fu, K. X., Sun, M., Yin, K., Wang, D., Liu, X., Crittenden, J. C. (2019). Phase-mediated heavy metal adsorption from aqueous solutions using two-dimensional layered MoS_2. ACS. Appl. Mater. Inter. *11*, 38789-38797. https://doi.org/10.1021/acsami.9b14019.

S17. Guo, J., Chen, T., Zhou, X. H., Zheng, T., Xia, W. N., Zhong, C. B., Liu, Y. C. (2019). Preparation and Pb (II) adsorption in aqueous of 2D/2D g-C_3N_4/MnO_2 composite. Appl. Organomet. Chem. *33*, e5119. https://doi.org/10.1002/aoc.5119.

S18. Fu, W., Wang, X. Y., Huang, Z. Q. (2019). Remarkable reusability of magnetic Fe_3O_4-encapsulated $C_3N_3S_3$ polymer/reduced graphene oxide composite: A highly effective adsorbent for Pb and Hg ions. Sci. Total. Environ. *659*, 895-904. https://doi.org/10.1016/j.scitotenv.2018.12.303.

S19. Rouhani, F., Morsali, A. (2018). Fast and selective heavy metal removal by a novel metal-organic framework designed with in-situ ligand building block fabrication bearing free nitrogen. Chem-Eur. J. *24*, 5529-5537. https://doi.org/10.1002/chem.201706016.

S20. Li, X., Xing, J. L., Zhang, C. L., Han, B., Zhang, Y. H., Wen, T., Leng, R., Jiang, Z. H., Ai, Y. J., Wang, X. K.(2018). Adsorption of lead on sulfur-doped graphitic carbon nitride nanosheets: experimental and theoretical calculation study. ACS. Sustain. Chem. Eng. *6*, 10606-10615. https://doi.org/10.1021/acssuschemeng.8b01934.

S21.Fu, W., Huang, Z. (2018). One-pot synthesis of a two-dimensional porous Fe_3O_4/Poly($C_3N_3S_3$) network nanocomposite for the selective removal of Pb(II) and Hg(II) from synthetic wastewater. ACS. Sustain. Chem. Eng. *6*, 14785-14794. https://doi.org/10.1021/acssuschemeng.8b03320.
